# Impact of state weights on national vaccination coverage estimates from household surveys in Nigeria

**DOI:** 10.1016/j.vaccine.2020.05.026

**Published:** 2020-07-06

**Authors:** Tracy Qi Dong, Dale A. Rhoda, Laina D. Mercer

**Affiliations:** a Department of Biostatistics, University of Washington, 1705 NE Pacific St, Seattle, WA 98195, USA; b Biostat Global Consulting, 870 High Street, Worthington, OH 43085, USA; c Institute for Disease Modeling, 3150 139th Ave SE, Bellevue, WA 98005, USA

**Keywords:** Vaccination coverage, Household surveys, Survey weight, DPT and MCV coverage, HBR availability, Nigeria

## Abstract

National vaccination coverage estimates from household surveys are widely used in monitoring and planning of immunization programs. In Nigeria, survey-reported national coverage estimates have shown large fluctuations in the past few years. In this paper, we examine the impact of state-level survey weighting on Nigeria’s national vaccination coverage estimation. In particular, we focus three vaccination-related outcomes among children aged 12–23 months: the coverage of the third dose of diphtheria, pertussis, and tetanus vaccine (DPT3); the coverage of the first dose of measles-containing vaccine (MCV1); and the availability rate of home-based vaccination record (HBR). We compare the sample selection and weight assignment of three major survey programs in Nigeria, and show that considerable portions of the changes in survey-reported national coverage estimates can be explained by shifts in state-level weights. Our analysis demonstrates the importance of state weighting method in estimating aggregated national coverage figures and provides important context for interpreting changes in coverage estimates between surveys in the future.

## Highlights

• Nigeria’s national survey estimates of DPT3 and MCV1 coverage fluctuate greatly in recent years.
• Much of the variation results from differences in surveys weights, not coverage.
• Both USAID DHS and UNICEF MICS allow weights to vary from round-to-round.
• Nigeria’s National Nutrition & Health Survey weights do not vary much due to post-stratification.
• To compare results from surveys proximate in time, use similar strata weights for clarity.

## 1 Introduction

Vaccination coverage is an important indicator of child health and access to health care [1]. In low- and middle-income countries where administrative data can be unreliable [2], household surveys are the primary data sources for vaccination coverage estimates [3], [4]. Country health care agencies often rely on survey-reported estimates to monitor immunization program performance and assess future vaccine needs [3]. The World Health Organization (WHO) and the United Nations Children’s Fund (UNICEF) also review household survey reports annually to calibrate and establish the WHO and UNICEF estimates of national immunization coverage (WUENIC) [5], [6].

Nigeria measures vaccination coverage at state and national levels using three families of survey: the Demographic and Health Surveys (DHS), the Multiple Indicator Cluster Surveys (MICS) and the National Nutrition & Health Surveys (NNHS) [7]. When results are released from a new survey, policymakers and international funding partners hasten to compare the latest coverage estimates with those from earlier work. If trends are addressed in the new report, the authors from a single survey family tend to compare with earlier results from that same family, even though there may be more recent estimates from other families. This intra-family focus is appropriate because surveys from the same family will hopefully have fewer differences in survey design and implementation [8]. However, policy- and investment-oriented stakeholders cannot afford to compare only within a single family; they must decide what to make of results from all of the well-respected national surveys.

**Fig. 1:**
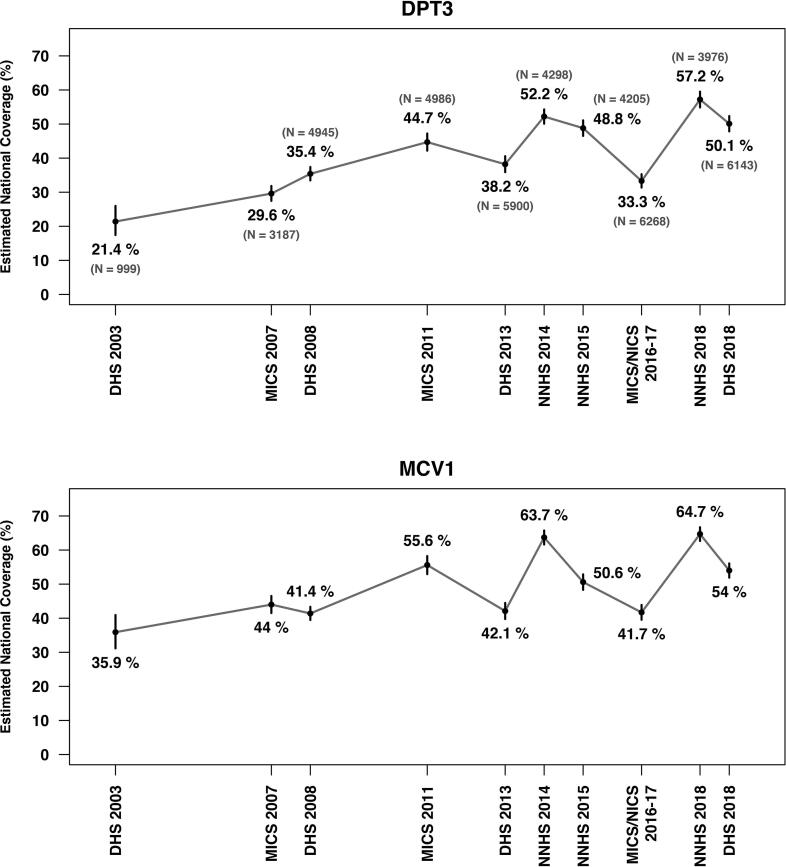
Survey-reported national DPT3 and MCV1 coverage estimates among children aged 12–23 months in Nigeria from 2003 to 2018. The vertical line segments indicate 95% confidence intervals, and the labels in parentheses list the number of children aged 12–23 months in the surveys. In all these surveys, vaccination status is based on evidence from either home-based records or caregiver recall.

In recent years, large fluctuations have been observed in Nigeria's survey-based national vaccination coverage estimates. Fig. 1 shows the survey-reported national coverage estimates of the third dose of diptheria, pertussis, and tetanus vaccine (DPT3) and the first dose of measles-containing vaccine (MCV1) among 12–23 month old children in Nigeria since 2003. To focus on a single example, the estimated national DPT3 coverage, which is commonly used to assess strength of routine immunization systems, dropped from 48.8% (95% CI: 46.5%51.1%) in 2015 [9] to 33.3% (95% CI: 31.0%35.1%) in 2016–17 [10]. This concerning development not only played an important role in motivating Nigeria’s National Primary Health Care Development Agency (NPHCDA) to declare a state of immunization emergency in 2017 [11], [12], but also provided a key data point for projecting future needs for vaccine supply and extending GAVI funding support [13], [14].

This paper examines data from all the DHS, MICS and NNHS in Nigeria since 2003 and annotates instances where national results for three important vaccination measures have appeared to improve or decline by more than ten percentage points. We decompose each of these apparent shifts into a portion which reflects apparent state-level trends and a portion which reflects changes in the proportion of the population that is attributed to each state (state weights). We show that this latter portion is surprisingly large in most of the double-digit differences, accounting for up to 80% of the apparent shift between consecutive surveys. The apparent shift would often have been much smaller if the earlier and later surveys had used the same state weights. This issue affects apparent differences *between and within* survey families, with DHS and MICS being more susceptible than NNHS.

In the sections that follow, we explore the impact of population distribution assumptions on national vaccination coverage survey estimates in Nigeria. First, we describe and compare the sample selection and weight calculation processes of the three survey families and review how national coverage estimates are calculated. Second, we examine apparent changes in three high-profile outcomes and identify eleven intra-family and nine inter-family shifts of ten or more percentage points in consecutive surveys. For each double-digit shift, we calculate the portion due to state-level outcome changes and the portion due to changes in state weights. We show how apparent coverage trends would differ if every survey used a common set of state weights. Finally, we discuss the implications of our analysis and make recommendations for clearer comparisons of consecutive surveys for any health outcome.

## 2 Background

### 2.1 Outcomes of interest

We examine apparent changes in three vaccination-related outcomes: (1) the proportion of children aged 12–23 months who showed a home-based vaccination record (HBR) to the survey interviewer; (2) the proportion of children aged 12–23 months who received DPT3; and (3) the proportion of children aged 12–23 months who received MCV1. HBR availability is conceptually simple and measured in the same way in DHS and MICS [15]. Measuring trends in DPT3 coverage is complicated because in 2012 Nigeria transitioned from a 3-antigen presentation to a pentavalent combination that also incorporates Hepatitis B and Haemophilus influenzae type B. There were concomitant changes in survey question wording and in caregiver awareness, knowledge, attitudes and preferences [16], [17], [18], [19]. Some biases involved in ascertaining whether a child received 3 doses of DPT will have surely changed in the time period of interest. Finally, measuring measles coverage is also complicated because measles vaccine is administered through both routine immunization activities and via periodic vaccination campaigns, also known as supplementary immunization activities. Surveys differ in how well they capture and analyze whether the child has had either a routine or campaign dose.

Measurement challenges that affect state-level outcome estimates are important, but they are not the focus of this paper. For our purposes, we aim to compare *observed changes in national coverage estimates between consecutive surveys* and *the change if both surveys were post-stratified using the same state weights*. This comparison reveals state weights as an important factor contributing to shifts in survey-reported national coverage estimates, above and beyond the apparent difference due to true underlying changes or sampling and non-sampling errors. We do not aim to establish an alternative estimate of the true national coverage levels for these outcomes. Instead, our objectives are to draw attention to an underappreciated and undocumented factor that has contributed significantly to large observed differences in national coverage estimates from consecutive surveys, and provide suggestions for policymakers to reduce this source of uncertainty when interpreting these trends.

### 2.2 Survey sampling

We consider a total of ten household surveys: four DHS conducted in 2003, 2008, 2013 and late 2018 [20], [21], [22], [23], three MICS conducted in 2007, 2011 and 2016–17^1^ [24], [25], [26], [10], [45] and three NNHS conducted in 2014, 2015 and early 2018 [27], [9], [28]. All the surveys used stratified cluster samples and estimated DPT3 and MCV1 coverage at the national level and in each of Nigeria’s 36 states and the Federal Capital Territory (FCT). (Hereafter we refer to these as *Nigeria’s 37 states*.) Both DHS and MICS also estimated HBR availability. The DHS samples were stratified by urban and rural areas within each state, whereas MICS and NNHS were stratified only by state. Respondents were selected in three stages: First, primary sampling units (PSUs or *clusters*) were selected from a sampling frame of enumeration areas (EAs) within each stratum. The 2003 DHS and 2007 MICS used frames derived from projections based on the 1991 census and the other surveys used frames derived from projections based on the 2006 census. Second, a pre-determined number of households were randomly selected in each cluster from a recently-updated frame listing every residential household. Third, field teams attempted to interview every eligible respondent from every selected household. DHS interviews women aged 15–49 years and collects vaccination data on their biological children under age 5 [29]. MICS interviews women aged 15–49 years and collects vaccination data on children under age 3 who are under their care in the household [29]. NNHS seeks vaccination data for all children under age 5 in the household.

### 2.3 Survey weighting

Every child in the dataset has an associated survey weight that quantifies the relative number of children he or she represents in the population. Broadly speaking the process of calculating weights involves some or all of three basic steps [30], [31], [32]. First, a base weight is calculated as the inverse of the respondent’s probability of selection. Second, base weights are optionally adjusted for non-response. If some selected households did not have anyone at home, or if some selected respondents refused to be interviewed, then the weight that would have been assigned to those non-respondents may be re-apportioned to respondents who were successfully interviewed. Third, if survey outcomes from more than one stratum are to be combined to form an aggregate coverage estimate, then the weights may be optionally *post-stratified*, i.e. the weights of all respondents in a stratum may be scaled by a multiplicative factor so that the sum of weights in every stratum is proportional to its eligible population. Table 1 describes how DHS, MICS and NNHS handled different components of sample selection and weight assignment. Here we briefly consider each component. Additional details are available in the supplementary materials.

**Table 1:** Elements of survey weights for vaccination coverage surveys in Nigeria.

Survey	Stratified by	First Stage Selection of Clusters (Within Each Stratum)	Base Weight Calculated Suing Stage 1 & 2 Selection Probabilities	Adjust for Non-response	Post-stratify
DHS	Urban/rural within each state	Sometimes equal probability and sometimes probability proportional to census population	Yes	Yes	No
MICS	State	Equal probability	Yes	Yes	No
NNHS	State	Probability proportional to 2006 census population size	No, assumed to be equal	No	Yes, scaled to match population proportion by state

The base weight of a survey sample is generally calculated using the inverse of the product of the probability of selection at each stage [30]. For the surveys considered here, DHS has varied how it handles the probability of first-stage selection, using equal probability in 2003 and 2008 and using probability proportional to census-based population size in 2013 and 2018 [20], [21], [22], [23]. MICS assigned equal probability to every cluster within each stratum in stage 1 [24], [25], [10]. Both survey programs have tracked the probability of household selection based on the updated frame from stage 2. The base weights for the survey samples were calculated using the probabilities from the two stages and vary from cluster to cluster. In contrast, the NNHS selected clusters with probability proportional to their population size (based on the 2006 census) within each stratum in stage 1. The NNHS base weights were all set to 1 using the SMART survey standard assumption that probability of selection is equal for each child [33]. If Nigeria’s census EAs have the same relative populations that were observed in 2006, then calculations that use NNHS base weights would be accurate; but if the relative population has changed since 2006, then the probability of selection is not truly equal for every child, and equal base weights will not accurately reflect the relative number of children represented by each respondent.

Both DHS and MICS adjust base weights for non-response whereas NNHS does not [33]. If the proportion of households and eligible respondents who are successfully interviewed is the same in every cluster, then the adjustment does not have any effect and is not needed. If the proportion of successful interviews varies by cluster, then omitting the non-response adjustment means the NNHS weights could fail to account for the differential response rate.

In Nigeria (and in general), neither MICS nor DHS post-stratify weights to match a set of administrative population figures. They proceed with the assumption that stratum-level sums of non-response-adjusted base weights reflect the relative population of eligible respondents at least as well as the figures projected by the National Population Commission (NPopC). This means that the survey sample stochastically determines the relative sums of weights in each state.

In contrast, NNHS does post-stratify; the base weights of all children under age 5 in the NNHS sample were scaled within each state so the sum of their weights would match the state’s relative population projected by NPopC based on the 2006 census. For children sampled who are aged 12–23 months, the sums of NNHS weights roughly, but do not exactly, match the population proportions projected by NPopC, because the portion of children sampled by NNHS who are aged 12–23 months varies stochastically from state to state. Note, then, that the NNHS sum of weights for children aged 12–23 months is constrained to be very nearly proportional to the relative state populations in 2006 whereas the DHS and MICS sums of weights are determined by the probabilities of selection and response in the sample and not constrained to the 2006 (or any other administrative) population distribution. While the first two stages of weighting are important for addressing representativeness and bias in the survey, it is this third stage of calculation, namely post-stratification or its absence, that drives the interesting phenomenon we document in this paper. Because the sums of state weights for DHS and MICS are determined by the sample and vary randomly, it appears that the portion of Nigeria’s 12–23 months population who live each state also varies randomly, and that variation can contribute to apparent outcome differences in a stealthy way.

### 2.4 Coverage estimation

With survey data, vaccination coverage estimates are commonly obtained using design-based statistical inference methods such as the Horvitz-Thompson (HT) estimators [34]. For a given vaccine, the point estimate of the national vaccination coverage (denoted as p^) can be written as

(1) p^=∑i=137∑j=1niwijyij∑i=137∑j=1niwij,

where *i* is the index for Nigeria’s 37 states, *j* is the index for children aged 12–23 months sampled in the survey, $n_{i}$ is the sample size in state $i,w_{\mathrm{ij}}$ is the weight associated to child *j* sampled in state *i*, and $y_{\mathrm{ij}}$ is a 0/1 indicator of whether child *j* in state *i* had received the vaccine at the time of interview. Re-arranging terms, we can re-write p^ as:

(2) p^=∑i=137∑j=1niwijyij∑j=1niwij⏟state-levelestimatei×∑j=1niwij∑i=137∑j=1niwij⏟stateweighti

Here, we use the term *state-level estimate* to represent the HT estimator of the vaccination coverage for a state, and the term *state weight* to represent the proportion of a state’s total weight to the sum of all states’ weights. Recall that a child’s survey weight represents the relative number of children he or she represents in the population. Therefore, the state weights reflect how the survey assumes the population of children aged 12–23 months are allocated across Nigeria’s states.

Eq. (2) shows that the national vaccination coverage estimate is a weighted aggregation of the state-level estimates. Therefore, any difference in national coverage estimate between two surveys can be partially explained by differences in state-level estimates and partially explained by differences in state weights.

## 3 Methods

We conducted two analyses to illustrate the impact of state weights on survey-reported national vaccination outcome estimates.

### 3.1 DPT3 and MCV1 Coverage Estimates from 2015 to 2016–17

From 2015 NNHS to 2016–17 MICS/NICS, the survey-reported point estimates of the national DPT3 and MCV1 coverage dropped from 48.8% to 33.3% and from 50.6% to 41.7%, respectively. We obtained the microdata of the two surveys and calculated the state-level coverage estimates of DPT3 and MCV1 using the survey package [35] within the R computing environment [36]. The *xlogit* option was used to compute the confidence intervals.^2^ We computed state weights and national coverage estimates using Eq. (2) and confirmed that our coverage results match the official observed values from survey reports up to the highest precision provided.^3^

We then post-stratified each survey at the state level using the population proportion by state from the 2006 census. This post-stratification step would not affect the state-level coverage estimates, because it scaled all weights within a state up or down by the same factor. This factor canceled out in the first term of Eq. (2) (see the supplementary materials for additional details). The resultant national coverage estimates would reveal one perspective on how much change there would be if the state weights were kept consistent between the two surveys. The discrepancy between this change and the observed change would help us understand how state weights contribute to the observed differences in national coverage estimates from consecutive surveys.

### 3.2 Double-digit shifts between consecutive surveys

We expanded the analysis in Section 3.1 to study all surveys conducted in Nigeria between 2003 and 2018. For each survey, we obtained the microdata and calculated the observed and post-stratified national estimates of DPT3 and MCV1 coverage. For MICS and DHS we also calculated HBR availability rate. Post-stratification was carried out at the state level using the total population proportions by state based on the 2006 census. We confirmed that the observed estimates from our calculation match the official survey reports up to the highest precision provided.

We identified every instance where a national outcome is observed to shift by more than ten percentage points between consecutive surveys both within and between survey families. We also identified instances where the post-stratified national outcome has a double-digit shift between consecutive surveys and compared the frequency of such instances with the observed trajectories. For each double-digit observed difference between two consecutive surveys, we calculated the *relative difference due to state weights* (*RDSW*). See the supplementary materials for details on this calculation.

Additionally, because vaccination outcomes can have some distracting measurement challenges, we also considered *faux* state-based survey outcomes — the latitude and longitude of the geographic center of each state — to help discern how the geographical aspects of the state weights vary across surveys. In particular, we examined how the 2016–17 MICS/NICS state weights compare with the other nine surveys of interest. Details of this analysis are provided in the supplementary materials.

## 4 Results

### 4.1 DPT3 and MCV1 Coverage Estimates from 2015 to 2016–17

**Fig. 2:**
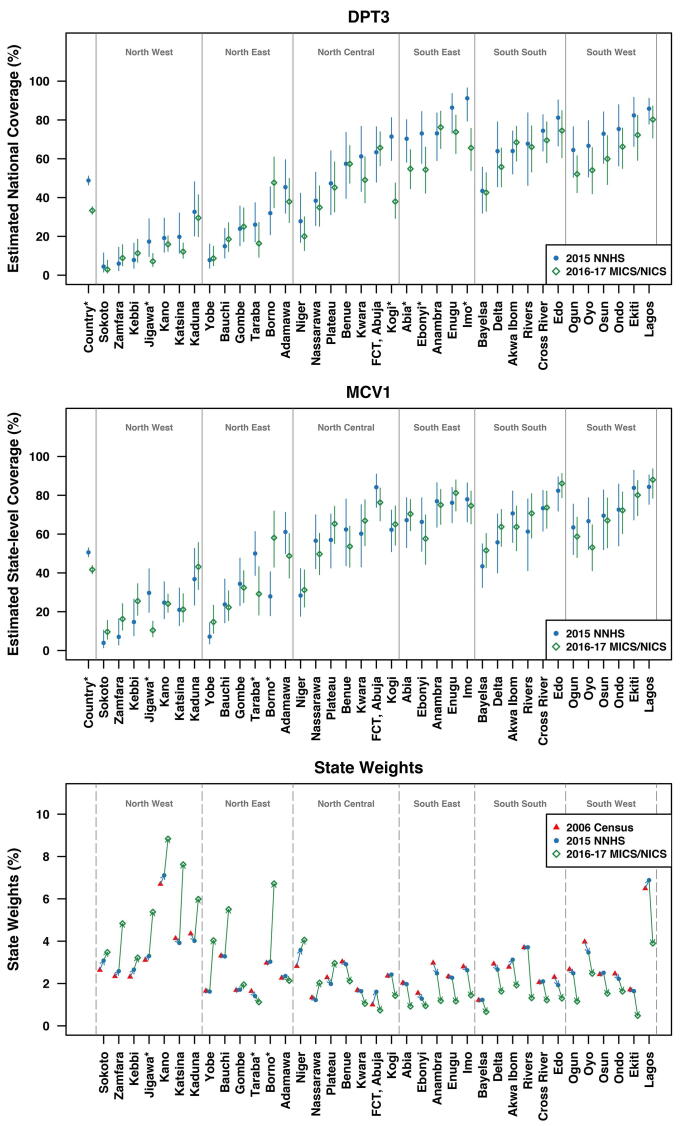
Top and middle: State-level DPT3 and MCV1 coverage estimates with 95% design-based confidence intervals in 2015 NNHS and 2016–17 MICS/NICS. Vaccination status is based on evidence from either home-based records or caregiver recall. States marked with asterisk∗ have statistically significant difference in coverage estimates between the two surveys based on one-sided z-test on logit-transformed coverage estimates at 95% significance level. Bottom: Total population proportion by state based on the 2006 Nigeria Census and state weights in 2015 NNHS and 2016–17 MICS/NICS.

Fig. 2 shows the state-level DPT3 and MCV1 coverage estimates, along with the census-based population proportions and the state weights from 2015 NNHS and 2016–17 MICS/NICS for Nigeria’s 37 states. The states are grouped into 6 geopolitical zones and arranged from left to right in order of ascending 2015 NNHS DPT3 estimates. The national coverage estimates are plotted on the far left for reference. In general, the northern states had lower coverage estimates than southern states in both surveys for both vaccines. Most states (27 out of 37) had decreased point estimates of DPT3 coverage from 2015 to 2016–17, although the magnitudes of the drops are generally not as large as the drop in the national estimate. More than half of the states (19 out of 37) had increased point estimates of MCV1 coverage between the two surveys. As expected, the 2015 NNHS state weights are very similar to the census-based population proportions, because the NNHS post-stratify children’s weights at the state level using figures projected by NPopC based on the 2006 census. On the other hand, the 2016–17 MICS/NICS state weights are very different from the 2015 NNHS weights and census-based population proportions — most northern states have higher weights and most southern states have lower weights in 2016–17 MICS/NICS compared to the others.

**Fig. 3:**
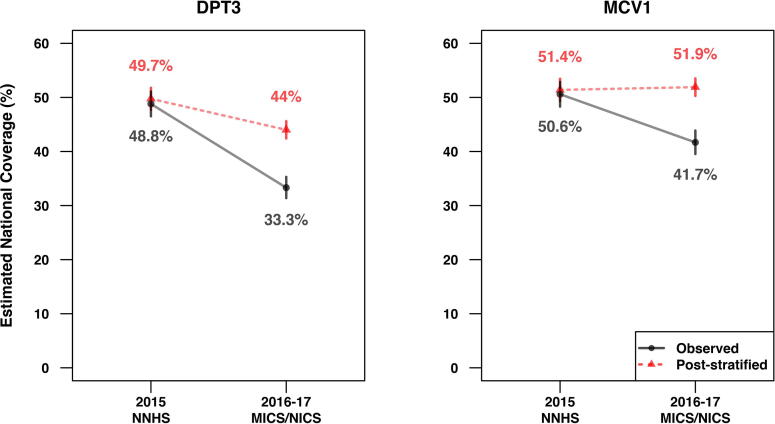
The observed national DPT3 and MCV1 coverage estimates from 2015 NNHS and 2016–17 MICS/NICS, and the post-stratified estimates after re-aggregating the state-level coverage estimates from each survey using the same population proportions based on the 2006 Nigeria Census. The vertical line segments indicate 95% confidence intervals.

The effects of the apparent differences in state weights are clearly shown in Fig. 3. The dots with the solid line indicate the observed national DPT3 and MCV1 coverage estimates, and the triangles with the dashed line indicate the post-stratified estimates after re-aggregating the state-level coverage estimates from each survey using the same census-based population proportions. The 2015 NNHS national estimates barely changed after post-stratification, but the re-aggregated 2016–17 MICS/NICS national estimates are much higher than the observed estimates. In this case, using the same set of state weights mitigates much of the drastic observed decrease in DPT3 coverage estimates between the two surveys. Notably, the observed decrease in MCV1 coverage from 2015 to 2016–17 actually reverses direction, resulting in a slight increase in the post-stratified estimates between the two surveys. These results demonstrate the potentially substantial contribution of state weight differences to apparent shifts in national coverage estimates across surveys.

### 4.2 Double-digit shifts between consecutive surveys

**Fig. 4:**
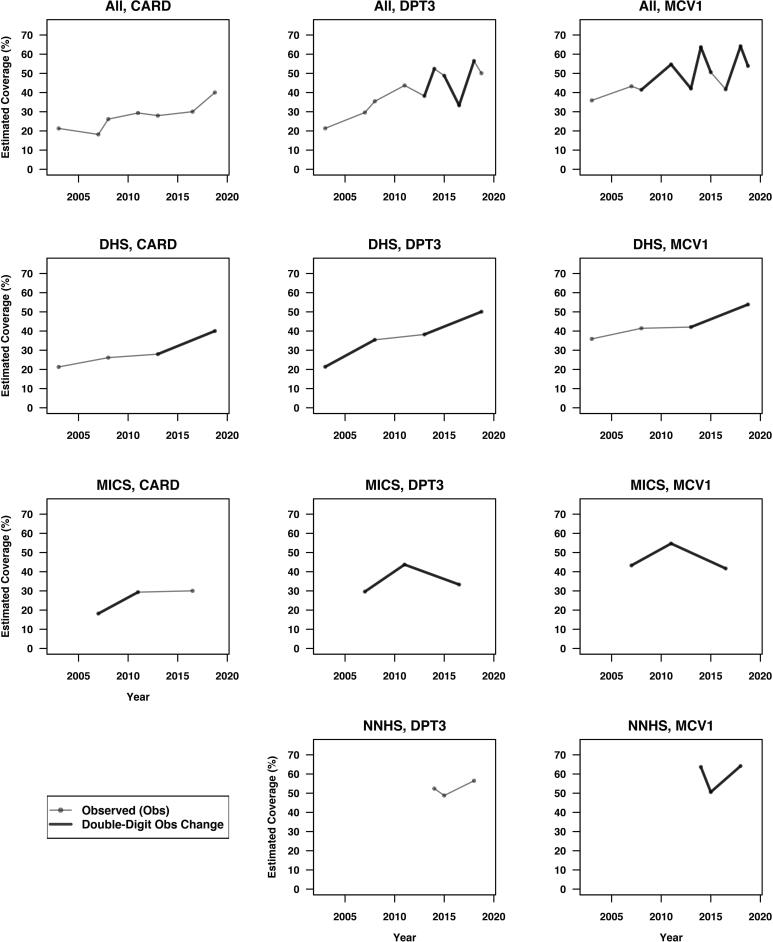
Trajectories of the observed national estimates of HBR availability rate (CARD), DPT3 and MCV1 coverage across surveys within and between survey families. All instances of double-digit shifts are highlighted using bold solid line segments between surveys.

**Fig. 5:**
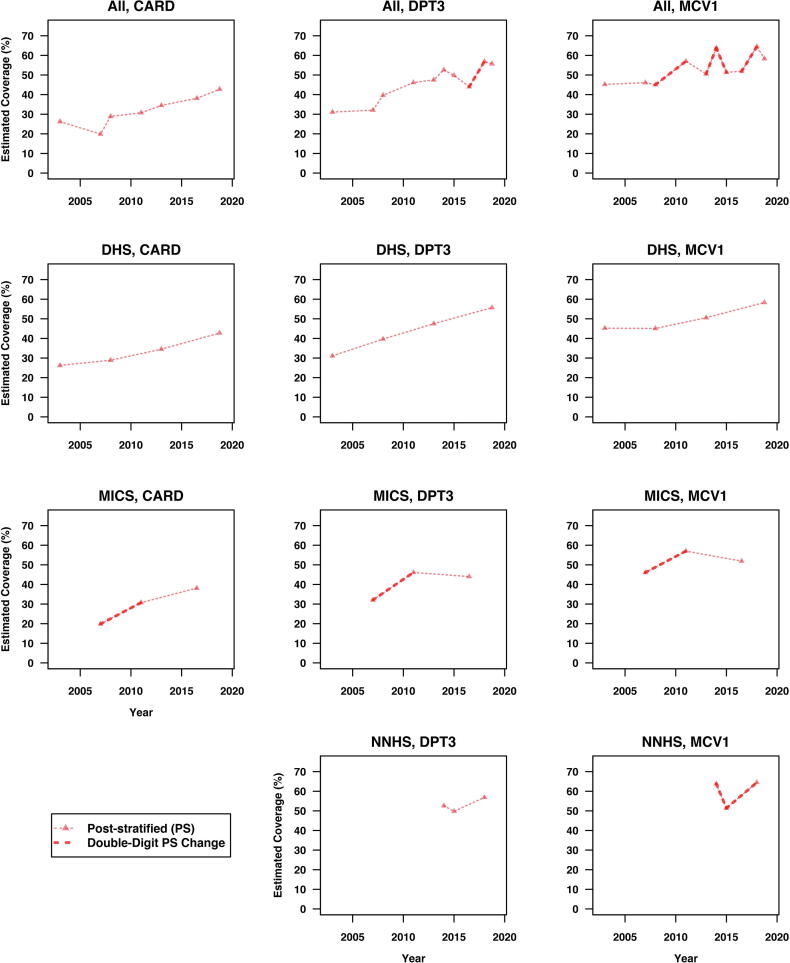
Trajectories of the post-stratified national estimates for HBR availability rate (CARD), DPT3 and MCV1 coverage across surveys within and between survey families. Post-stratification is carried out at the state level using the total population proportions by state based on the 2006 Census. All instances of double-digit shifts are highlighted using bold dashed line segments between surveys.

**Fig. 6:**
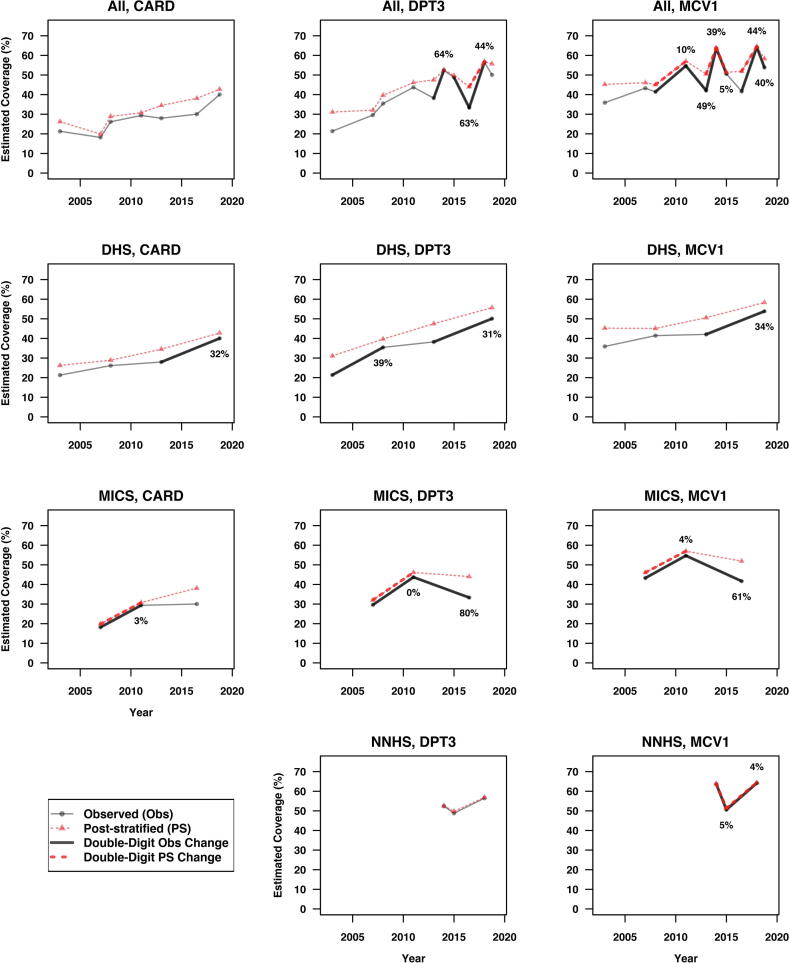
Trajectories of the observed and post-stratified national estimates for HBR availability rate (CARD), DPT3 and MCV1 coverage across surveys within and between survey families. Post-stratification is carried out at the state level using the total population proportions by state based on the 2006 Census. All instances of double-digit shifts are highlighted using bold solid/dashed line segments between surveys. The *RDSW* values for all instances where the observed estimates experience double-digit shifts are annotated accordingly.

Fig. 4, Fig. 5 show the trajectories of the observed national estimates and post-stratified national estimates for HBR availability rate (CARD), DPT3 and MCV1 coverage across surveys within and between survey families. All instances of double-digit shifts are highlighted using bold line segments between surveys. Fig. 6 overlays Fig. 4, Fig. 5, and annotates the *RDSW* values for all instances where the observed estimates experience double-digit shifts.

The top row of Fig. 6 shows the shifts when surveys are assessed in chronological order across families. DPT3 had three double-digit shifts that are 44%, 63% and 64% due to differing state weights. MCV1 has a sawtooth pattern with six double-digit shifts, two being minorly due to weights (5% and 10%) and four where 39–49% are attributable to differing state weights.

Within NNHS (bottom row), we see that *RDSW* is small for the two double-digit observed shifts. This is not a surprise because NNHS post-stratifies its children sample to the same state totals each year. As expected, we see nearly all of the NNHS observed differences to be due to changes in state-level outcomes. Within DHS, there are four double-digit observed shifts. They appear in all three outcomes, and changes in weights account for 30–40% of the shifts in every case. Within MICS, there are also double-digit shifts for every outcome. Three of the *RDSW* are very small and two relating to the 2016 MICS are very large. Four-fifths of the apparent 10% drop in DPT3 coverage from 2011 to 2016 is due to differences in the state weights, as is three-fifths of the apparent 13% drop in MCV1 over the same time period.

Post-stratification in Fig. 5, Fig. 6 eliminates 8 of the 20 apparent double-digit shifts from Fig. 4 and does not introduce any new ones. Post-stratification smooths the saw-tooth patterns in observed differences; the highest outcomes do not appear to shift, but the lowest estimates are revised notably upward. If we standardized with a different set of state weights, the results could change.

## 5 Discussion

We were initially drawn to examine DPT3 coverage because the observed 15.5% decrease to 33.3% national DPT3 coverage estimate reported in the 2016–17 MICS/NICS motivated action and mobilized resources. It was influential in NPHCDA’s declaration of a state of immunization emergency [11], [12] and in GAVI funding support extension [13], [14]. Our analysis revealed that much of this aggregate national shift could be attributed to differences in state weights. This is not to suggest that there was not or is not currently a state of emergency with respect to routine immunization in Nigeria — regardless of which weights we use for aggregation, the national DPT3 coverage estimate is substantially below the 90% target of the Global Vaccine Action Plan [39] and requires immediate improvement. However, we aim to emphasize the need for better understanding of observed changes in national estimates across surveys and identifying the contribution of state weights when interpreting these trends.

At the helpful suggestion of an anonymous reviewer, we broadened our focus to include additional outcomes and decided to examine all the double-digit shifts. That broader examination revealed that differences in state weights contribute considerably to apparent shifts both within and between survey families and across all three outcomes considered here. These analyses serve as a strong reminder for policy-makers to interpret and compare survey-reported national coverage estimates with caution. We do not think the issue is limited only to vaccination outcomes or only to children, so the topic is a good candidate for additional investigation in Nigeria and elsewhere.

One should note that even if two surveys use a common set of state weights, their results are still not perfectly comparable. Many aspects of survey design and implementation contribute to differences in net biases of the state-level estimates. Those aspects include differences in: survey protocols; question wording, question order, or response options; rates of HBR availability versus caregiver recall; and different data collection teams who undergo different training regimens and are subject to different systems of monitoring and supervision. The magnitude of these biases are not easily quantifiable. Even if a common set of state weights were employed in trend analyses, policy-makers and donor partners would still need to read survey reports carefully to decide how much credence to give apparent shifts in aggregated outcomes.

So what is the conscientious survey analyst to do? The most recent census in Nigeria was 14 years ago and other countries face the same challenge. Shall we assume that the population is frozen in time and distributed as it was when last counted? If the relative populations of the states are different now, then applying weights from the last census would introduce bias into new national estimates. What is wanted is an up-to-date, widely respected, officially endorsed estimate of the relative population of the states. One possibility would be to use recent gridded population estimates like those from FlowMinder and WorldPop that integrate remote sensing data with mobile phone cell tower data to build so-called *bottom-up* population estimates [40], [41], [42]. It would be instructive to evaluate gridded datasets for the years considered here to understand how those population estimates change from 2006 to 2018 and how they compare with the survey-derived estimates. Another solution would be for governments or influential funding partners to insist that the surveys used for official national estimates use officially endorsed state weights. This would introduce bias that changes over time, but would ensure that apparent changes were based entirely on state-level outcomes rather than poorly documented and poorly understood changes in weights.

Consumers of these surveys are at a disadvantage because even if stakeholders appreciated weights as a possible source of error, survey reports document the weight calculation method, but do not usually document how weights are allocated across states. (Table 1.6 in [43] is an exception.) It would be impossible for the most sophisticated reader of the ten survey reports represented here to identify how the weights in a new survey compared with those in previous surveys in the same family or other families. Without independent analysis of the microdata, there is no straightforward way for a reader of the 2016–17 MICS report to discern that 80% of the MICS 2011 to 2016–17 drop in DPT3 is due to differences in weights and 20% is due to state-level outcomes. We offer the following suggestions to address this disadvantage.

1. Survey reports could list the relative sums of weights in an annex, showing what portion of the eligible population is represented by the weights in each stratum. The portions should sum to 100%.
2. The same annex could indicate how those population proportions differ from those in the most recent census.
3. The same annex could indicate whether the state weights differ from those used in the last survey in this same family, and if yes, by how much.
4. Where possible, it would be a kind service to also indicate whether the state weights differ from those in recent surveys from other families that report the same outcomes, and if yes, by how much.
5. When important outcomes appear to shift by a notable amount, maybe five or ten percentage points from the previous survey in the same family, the report usually comments on the apparent shift. Perhaps the report could also comment on how much the outcome would have shifted if the two datasets had been analyzed with a common set of weights. This suggestion is mildly complicated by the fact that the details will depend on *which* set of common weights are employed. Using the weights produced by the most recent census, or the most recent officially endorsed set of weights probably makes sense. It would also be possible to use the weights from the earlier of the two surveys as the common set of weights. (In results not shown here, we found that our estimates of *RDSW* were quite similar if we post-stratified using: (a) the 2006 census all-ages population, (b) the 2006 census under-age-5 population, or (c) the weights that were employed in the earlier survey.)
6. The WHO/ UNICEF WUENIC process of synthesizing vaccination coverage outcomes commonly makes an adjustment to survey data to align vaccination dropout documented by caregiver recall with dropout documented on home-based and facility-based records [44]. In other words, WUENIC changes the reported survey outcome to adjust for a well-known bias concerning caregivers’ propensity to under-report receipt of doses that fall late in a series. It might be reasonable for WUENIC to also adjust reported survey outcomes to use a set of state weights that are officially endorsed.

Note that suggestions 1–5 above are germane for any survey outcome; they are not specific to vaccination or even to children. For examples of summaries that could be provided in the survey reports, see the supplementary materials.

As mentioned in Section 2.3, DHS and MICS in Nigeria do not post-stratify state weights; instead the samples stochastically determine the state weights. In this sense, the dramatic northern population shift implied by the 2016–17 MICS/NICS sample may have been a statistical anomaly. Alternatively, it is possible that the 2016–17 MICS/NICS detected an as-yet undocumented northward shift in population in Nigeria. However, that seems unlikely, considering the 2018 DHS state weights are reasonably close to the 2006 census figures. (See the supplement to visualize the northward shift.).

Finally, our analyses may partially explain why national vaccination coverage estimates from NNHS are often higher than those from other surveys. Recall that NNHS post-stratify the children’s weights to match the relative *total population* in each state as projected by NPopC. This process carries an implicit assumption that the children’s population proportions are similar to the total population proportions by state. However, because of the difference in population age structure across states, this assumption does not hold, resulting in less weight assigned to the children sampled in northern states and more weight assigned to the children sampled in southern states. As such, the aggregated national coverage estimates will be higher since the southern states usually have higher coverage estimates. We suggest that future NNHS consider post-stratifying children’s weights using age-specific population proportions, instead of the total population proportions.

## 6 Conclusions

This study serves as the first analysis of the impact of differences in state-level weights between surveys on the national vaccination coverage estimates in Nigeria. Our analysis facilitates a holistic assessment of national coverage estimates from nationally representative household surveys conducted from 2003 to 2018 in Nigeria. Lastly, this approach provides important context for interpretation of changes in national coverage estimates between surveys in the future.

## 7 Funding

TQD and LDM were supported by Bill and Melinda Gates through the Global Good Fund while at the Institute for Disease Modeling. DAR was funded by the Bill & Melinda Gates Foundation (Investment IDs #29065 & #53009).

## Declaration of Competing Interest

The authors declare no known conflict of interest.

## Footnotes

1 The 2016–17 MICS is combined with the National Immunization Coverage Survey (NICS).

2 This is often named the *logit* method in survey proportion literature [37], [38] but is called *xlogit* in R’s survey package.

3 The 2016–17 MICS/NICS calculated weights for the nationally representative survey as well as alternative weights for state-specific reports in Kano and Lagos. Only the weights for the nationally representative survey were used in this analysis.
