# Supplementary material for: Impact of state weights on national vaccination coverage estimates from household surveys in Nigeria
Source: Vaccine. 2020 Jul 6;38(32):5060–70. doi: 10.1016/j.vaccine.2020.05.026 (PMC7327524; doi:10.1016/j.vaccine.2020.05.026)
Supplement: Supplementary file 1 [file mmc1.pdf]

Supplementary materials for "Impact of state weights on national vaccination coverage estimates from household surveys in Nigeria"

Tracy Qi Dong[1], Dale A. Rhoda[2], Laina D. Mercer[3]

[1] Department of Biostatistics, University of Washington, Health Sciences Building, NE Pacific St, Seattle, WA 98195, USA
[2] Biostat Global Consulting, 870 High Street, Worthington, OH 43085, USA
[3] Institute for Disease Modeling, 3150 139th Ave SE, Bellevue, WA 98005, USA

1 Weight calculation breakdown

We break down the weight calculation for each survey program to show the various components we discussed in Section 2 of the main paper.

1.1 DHS weight calculation

In general, the weight associated to each child in cluster c of stratum s in a DHS survey can be calculated as

$$W_{sc} = \frac{M_s}{m_s} \times \frac{H_{sc}}{h_{sc}} \times \frac{1}{r_s^{(h)} r_s^{(w)}}$$

where

- M_s = the total number of clusters in stratum s according to the frame.
- m_s = the number of clusters sampled in stratum s.
- H_{sc} = the total number of households listed in cluster c of stratum s after listing is completed.
- h_{sc} = the number of households sampled in cluster c of stratum s.
- $r_s^{(h)}$ = the household response rate in stratum s.
- $r_s^{(w)}$ = the women response rate in stratum s.

Exact weight calculation varies from survey to survey depending on specific details in the sampling design. Although survey reports discuss using probability proportional to size (PPS) for cluster selection, many recent DHS surveys used simple random sample (SRS) in the cluster selection stage. For surveys that actually used PPS, the individual child weight can be calculated as

$$W_{sc} = \frac{N_s}{m_s N_{sc}} \times \frac{H_{sc}}{h_{sc}} \times \frac{1}{r_s^{(h)} r_s^{(w)}}$$

where

- N_s = the total population of stratum s according to the frame.
- N_{sc} = the total population of cluster c of stratum s according to the frame.

1.2 MICS weight calculation

In general, the weight associated to each child in cluster c of stratum s in a MICS survey can be calculated as

$$W_{sc} = \frac{M_s}{m_s} \times \frac{H_{sc}}{h_{sc}} \times \frac{1}{r_s^{(h)} r_s^{(k)}}$$

where $r_s^{(k)}$ is the children response rate in stratum s

Although technical document on MICS design and sampling discusses using PPS for cluster selection, all the MICS surveys we examine in this paper used SRS in the cluster selection stage.

1.3 NNHS weight calculation

In general, the weight associated to each child in each cluster of stratum s can be calculated as

$$W_s = 1 \times g_s$$

where g_s is the state-level post-stratification factor applied to the based weight, such that the final state weights after post-stratification match the population distribution figures provided by NPopC based on the 2006 census. It should be noted that the "self-weighting" in the NNHS surveys are carried out by ignoring weighting and simply assigning everyone to have weight 1. Therefore, post-stratification is necessary.

2 Post-stratification

We post-stratify each survey at the state level using the population proportion by state from 2006 Nigeria Census. Specifically, the survey weight associated to each child, w_{ij} is multiplied by the same multiplicative factor $g_i = N_i/N$ **within each state**, $w^*_{ij} = w_{ij} \times g_i$. Where N_i is the population of state i according to the 2006 Nigeria Census and N is the overall population. Mathematically, the national coverage estimate in a survey after post-stratification is

$$\hat{p}^{PS} = \sum_{i=1}^{37} \left(\underbrace{\left[\frac{\sum_{j=1}^{n_i} w^*_{ij} y_{ij}}{\sum_{j=1}^{n_i} w^*_{ij}} \right]}_{\text{state-level estimate } i} \times \underbrace{\left[\frac{\sum_{j=1}^{n_i} w^*_{ij}}{\sum_{i=1}^{37} \sum_{j=1}^{n_i} w^*_{ij}} \right]}_{\text{post-stratified state weight } i} \right) = \sum_{i=1}^{37} \left(\underbrace{\left[\frac{\sum_{j=1}^{n_i} g_i w_{ij} y_{ij}}{\sum_{j=1}^{n_i} g_i w_{ij}} \right]}_{\text{state-level estimate } i} \times \underbrace{\left[\frac{\sum_{j=1}^{n_i} g_i w_{ij}}{\sum_{i=1}^{37} \sum_{j=1}^{n_i} g_i w_{ij}} \right]}_{\text{post-stratified state weight } i} \right) \tag{1}$$

$$= \sum_{i=1}^{37} \left(\underbrace{\left[\frac{g_i}{g_i} \times \frac{\sum_{j=1}^{n_i} w_{ij} y_{ij}}{\sum_{j=1}^{n_i} w_{ij}} \right]}_{\text{state-level estimate } i} \times \underbrace{\left[\frac{\sum_{j=1}^{n_i} g_i w_{ij}}{\sum_{i=1}^{37} \sum_{j=1}^{n_i} g_i w_{ij}} \right]}_{\text{post-stratified state weight } i} \right) = \sum_{i=1}^{37} \left(\underbrace{\left[\frac{\sum_{j=1}^{n_i} w_{ij} y_{ij}}{\sum_{j=1}^{n_i} w_{ij}} \right]}_{\text{state-level estimate } i} \times \underbrace{\left[\frac{\sum_{j=1}^{n_i} g_i w_{ij}}{\sum_{i=1}^{37} \sum_{j=1}^{n_i} g_i w_{ij}} \right]}_{\text{post-stratified state weight } i} \right) \tag{2}$$

Note that post-stratification does not affect the state-level coverage estimates, because it scales all weights within a state up or down by the same factor, which cancels out in the first term of Equation (1). However, this does affect the state weights and the aggregate national estimate, because the common factor remains in the second term of Equation (1) as shown in Equation (2) and shifts the portion of the whole that is represented by state i.

3 Calculation of the Relative Difference due to State Weights

For each double-digit observed difference between two consecutive surveys, we calculate the *relative difference due to state weights (RDSW)*:

$$RDSW_{kl} = 100 \times \frac{(\hat{p}_k - \hat{p}_l) - (\hat{p}_k^{PS} - \hat{p}_l^{PS})}{(\hat{p}_k - \hat{p}_l)} \quad (3)$$

where k denotes the earlier survey, l denotes the later survey, $\hat{p}$ denotes the observed national estimate calculated using the survey's original state weights, and $\hat{p}^{PS}$ denotes the post-stratified national estimate calculated using the consistent census-based population proportions. It should be noted that we use $RDSW$ in this particular analysis to measure the portion of the observed difference between consecutive surveys attributable to differing state weights — it may not be an appropriate summary in every setting because $RDSW$ is not defined if the denominator is zero and might be difficult to interpret if a) the denominator is very small, b) the common-weight difference exceeds the original or c) the common-weight difference reverses direction from the original. In many situations, it will be clearest to simply report the original difference $(\hat{p}_k - \hat{p}_l)$ and the common-weight difference $(\hat{p}_k^{PS} - \hat{p}_l^{PS})$.

4 Example Tables Showing Changes in State Weights

In the discussion section of the main manuscript we suggest that nationally representative surveys might consider adding a report annex to describe state weights. This supplement provides a mock-up of what that annex might have looked like for children age 12–23 months in eight of the ten surveys described in the manuscript.

4.1 Methods

For the eight surveys described in our manuscript that used a sampling frame based on the 2006 census, we obtained the microdata and summed the survey weights for children aged 12–23m in every state. We divided each state-level sum by the overall sum to obtain the so-called state-weight, which is the portion of Nigerian 12–23m old children assumed to live in each state.

Each table on the following pages arranges the 37 states of Nigeria, starting with states in the northern zones followed by states in southern zones. Within each group, the states are sorted in order of their Census 2006 state weights. Each table shows five columns of numbers:

(a) the Census 2006 state weights (for all ages)

(b) the new survey state weights (children age 12-23 months)

(c) the simple difference between the new survey and census state weights

(d) the simple difference between the new survey state weights and the state weights from the previous survey from the same survey family (where applicable). That is, if the new survey is a DHS, show the difference between the state weights for the new DHS and the previous DHS; if it is a MICS, show the difference between the new MICS and the previous MICS; same for NNHS.

(e) the simple difference between the new survey state weights and the weights for the previous survey, regardless of whether the previous survey is a DHS, MICS or NNHS

Each number is reported to one decimal place and accompanied by a colored bar graph: blue bars for weights; green for positive differences; red for negative differences.

4.2 Results

There are interesting differences represented on nearly every page – either differences with the census, the previous survey in the same family, or the previous survey in another family. Differences between surveys in consecutive years, or the same year are especially unlikely to reflect true changes in population distribution.

- DHS 2008 was the first of these surveys to use the 2006 census as a sampling frame. Its weight for children age 12–23m in the state of Bauchi was much lower than that of DHS 2003. The state-level differences between DHS 2008 and MICS 2007 (only one year before) are notable with absolute value $\geq 1.0\%$ in 13 states.

- MICS 2011 shows some notable differences with MICS 2007 and differences $\geq 1.0\%$ with DHS 2008 for 6 states.

- DHS 2013 shows a pattern of giving additional weight to northern states compared with the census and compared with the previous DHS and the previous MICS. Several differences have magnitude $> 2.0\%$.

- NNHS 2014 uses state weights very close to the 2006 census. Compared with the preceding DHS 2013, it gives substantially more weight to southern states than northern states, which probably helps explain why its vaccination coverage estimates were higher than those from DHS. (See Figures 4-6 in the main manuscript.)

- NNHS 2015 is very similar to NNHS 2014.

- MICS-NICS 2016 shows a notable pattern of high weights in the north and low weights in the south compared with the census, the previous MICS, and the NNHS from one year prior.

- NNHS 2018 is post-stratified, so is very similar to NNHS 2015, except for Kano & Borno. NNHS 2018 is markedly different from MICS-NICS 2016.

- DHS 2018 shows more weight in the north and less in the south compared with the census, but not a broad pattern of shift from the last DHS.

4.3 Strengths & Limitations

Our suggestion to add such a summary to reports that aggregate results across strata is simple to implement, especially for looking at differences within a survey family. Reports from one survey family do not usually acknowledge other families, so it may not be realistic to think that all families would agree to include the final, right-most column in each of these tables.

These tables were constructed with the benefit of hindsight, so were stratified by northern and southern states, which accentuates some of the structure in this dataset. In a production setting, the states would be more likely to be listed in alphabetical order, which would carry the same information as our tables, but might make patterns less obvious.

We have only shown the tables for children age 12-23 months, which is the reporting standard for vaccination coverage outcomes. A DHS or MICS or NNHS report includes numerous indicators for numerous age groups, so presumably this suggestion would not result in a single table per report, but perhaps one table for each indicator-relevant demographic age group; it might be five or six tables per

report rather than one. And in some cases the age ranges for different survey families would be slightly different, so the tables would not always be strictly comparable across survey families, but the overall idea here is to make the changes in survey weights obvious to the interested reader so they can ask an analyst to drill down with the microdata and decompose the portion of apparent shifts due to changes in state weights. If the tables show whether the weights were substantially the same as those in the census or the previous survey(s), they will likely provide enough of a clue to spark those detailed analyses.

4.4 Conclusion

It would be straightforward to incorporate tables like these into the annexes of survey reports where results are aggregated across strata to furnish a nationally representative estimate of important health indicators.

	State	Census 2006	DHS 2008			
			State weight	Census 2006 Difference	DHS 2003 Difference	MICS 2007 Difference
North	Kano	6.7%	8.0%	1.3%	-1.0%	-1.8%
	Kaduna	4.4%	5.0%	0.7%	-0.7%	-2.9%
	Katsina	4.1%	5.4%	1.3%	-0.5%	2.6%
	Bauchi	3.3%	4.0%	0.7%	-4.5%	-0.3%
	Jigawa	3.1%	3.8%	0.7%	0.6%	-0.2%
	Benue	3.0%	2.7%	-0.3%	-0.8%	-0.3%
	Borno	3.0%	3.6%	0.6%	-0.2%	0.0%
	Niger	2.8%	2.8%	0.0%	-1.8%	1.2%
	Sokoto	2.6%	3.6%	1.0%	0.2%	1.6%
	Kogi	2.4%	1.6%	-0.8%	-0.9%	0.5%
	Zamfara	2.3%	3.0%	0.6%	-1.1%	1.2%
	Kebbi	2.3%	2.6%	0.3%	-1.7%	0.5%
	Plateau	2.3%	2.2%	0.0%	0.3%	0.5%
	Adamawa	2.3%	2.4%	0.2%	1.1%	0.7%
	Kwara	1.7%	1.4%	-0.3%	0.8%	0.3%
	Gombe	1.7%	2.0%	0.3%	0.2%	0.3%
	Yobe	1.7%	2.1%	0.4%	-0.9%	-0.5%
	Taraba	1.6%	1.8%	0.2%	-1.6%	0.1%
	Nassarawa	1.3%	1.1%	-0.3%	-0.2%	-0.4%
	FCT Abuja	1.0%	1.0%	0.0%	0.5%	0.6%
South	Lagos	6.5%	5.3%	-1.2%	1.9%	-2.9%
	Oyo	4.0%	3.6%	-0.4%	2.1%	-1.4%
	Rivers	3.7%	4.2%	0.5%	1.0%	1.9%
	Anambra	3.0%	3.1%	0.1%	0.4%	1.8%
	Delta	2.9%	2.0%	-0.9%	-0.7%	-1.2%
	Imo	2.8%	2.2%	-0.6%	0.0%	0.4%
	Akwa Ibom	2.8%	2.0%	-0.7%	-0.1%	-1.3%
	Ogun	2.7%	2.5%	-0.2%	1.2%	0.2%
	Ondo	2.5%	1.8%	-0.6%	1.1%	0.1%
	Osun	2.4%	1.7%	-0.8%	1.1%	-1.4%
	Enugu	2.3%	1.9%	-0.4%	0.6%	0.0%
	Edo	2.3%	2.0%	-0.3%	0.0%	-0.2%
	Cross River	2.1%	2.1%	0.0%	0.4%	-0.5%
	Abia	2.0%	1.4%	-0.6%	0.9%	-0.2%
	Ekiti	1.7%	1.6%	-0.1%	0.9%	0.7%
	Ebonyi	1.6%	1.5%	-0.1%	0.8%	-0.2%
	Bayelsa	1.2%	1.1%	-0.1%	0.8%	0.4%

Table W01. State weights for children age 12-23 months in DHS 2008, along with state-wise weight differences with: Census 2006, DHS 2003 and MICS 2007.

Region	State	Census 2006	MICS 2011 State weight	MICS 2011 Census 2006 Difference	MICS 2011 MICS 2007 Difference	MICS 2011 DHS 2008 Difference
North	Kano	6.7%	7.1%	0.4%	-2.7%	-0.9%
North	Kaduna	4.4%	4.4%	0.0%	-3.6%	-0.7%
North	Katsina	4.1%	4.4%	0.3%	1.6%	-1.0%
North	Bauchi	3.3%	5.1%	1.8%	0.8%	1.1%
North	Jigawa	3.1%	3.0%	-0.1%	-1.1%	-0.9%
North	Benue	3.0%	2.6%	-0.4%	-0.4%	-0.1%
North	Borno	3.0%	3.4%	0.5%	-0.1%	-0.1%
North	Niger	2.8%	2.8%	0.0%	1.1%	0.0%
North	Sokoto	2.6%	3.2%	0.6%	1.3%	-0.4%
North	Kogi	2.4%	1.8%	-0.6%	0.7%	0.2%
North	Zamfara	2.3%	2.9%	0.5%	1.1%	-0.1%
North	Kebbi	2.3%	2.8%	0.4%	0.7%	0.2%
North	Plateau	2.3%	1.9%	-0.4%	0.1%	-0.4%
North	Adamawa	2.3%	2.4%	0.1%	0.6%	-0.1%
North	Kwara	1.7%	1.8%	0.1%	0.7%	0.4%
North	Gombe	1.7%	2.0%	0.3%	0.3%	0.1%
North	Yobe	1.7%	1.9%	0.3%	-0.6%	-0.1%
North	Taraba	1.6%	1.4%	-0.2%	-0.3%	-0.4%
North	Nassarawa	1.3%	1.4%	0.0%	-0.1%	0.3%
North	FCT Abuja	1.0%	1.0%	0.0%	0.6%	0.0%
South	Lagos	6.5%	6.7%	0.2%	-1.6%	1.4%
South	Oyo	4.0%	3.7%	-0.2%	-1.2%	0.2%
South	Rivers	3.7%	3.1%	-0.6%	0.8%	-1.0%
South	Anambra	3.0%	2.5%	-0.5%	1.2%	-0.6%
South	Delta	2.9%	3.1%	0.2%	-0.1%	1.1%
South	Imo	2.8%	1.8%	-1.0%	0.0%	-0.4%
South	Akwa Ibom	2.8%	3.0%	0.3%	-0.3%	1.0%
South	Ogun	2.7%	2.4%	-0.3%	0.1%	-0.1%
South	Ondo	2.5%	2.3%	-0.1%	0.6%	0.5%
South	Osun	2.4%	2.2%	-0.3%	-0.9%	0.5%
South	Enugu	2.3%	2.3%	0.0%	0.4%	0.4%
South	Edo	2.3%	1.6%	-0.7%	-0.6%	-0.4%
South	Cross River	2.1%	2.1%	0.0%	-0.5%	0.0%
South	Abia	2.0%	1.9%	-0.1%	0.3%	0.5%
South	Ekiti	1.7%	1.4%	-0.3%	0.6%	-0.1%
South	Ebonyi	1.6%	1.4%	-0.2%	-0.3%	-0.1%
South	Bayelsa	1.2%	1.5%	0.2%	0.7%	0.4%

Table W02. State weights for children age 12-23 months in MICS 2011, along with state-wise weight differences with: Census 2006, MICS 2007 and DHS 2008.

Region	State	Census 2006	DHS 2013 State weight	DHS 2013 Census 2006 Difference	DHS 2013 DHS 2008 Difference	DHS 2013 MICS 2011 Difference
North	Kano	6.7%	9.0%	2.3%	1.0%	1.9%
North	Kaduna	4.4%	3.8%	-0.6%	-1.3%	-0.6%
North	Katsina	4.1%	5.4%	1.3%	0.0%	1.0%
North	Bauchi	3.3%	4.5%	1.2%	0.5%	-0.5%
North	Jigawa	3.1%	5.3%	2.1%	1.4%	2.3%
North	Benue	3.0%	3.2%	0.2%	0.5%	0.6%
North	Borno	3.0%	3.8%	0.8%	0.2%	0.3%
North	Niger	2.8%	4.4%	1.5%	1.5%	1.6%
North	Sokoto	2.6%	3.4%	0.8%	-0.2%	0.2%
North	Kogi	2.4%	1.4%	-1.0%	-0.2%	-0.4%
North	Zamfara	2.3%	4.9%	2.6%	2.0%	2.1%
North	Kebbi	2.3%	3.8%	1.4%	1.2%	1.0%
North	Plateau	2.3%	1.6%	-0.7%	-0.7%	-0.3%
North	Adamawa	2.3%	2.3%	0.0%	-0.2%	-0.1%
North	Kwara	1.7%	1.2%	-0.5%	-0.2%	-0.6%
North	Gombe	1.7%	1.7%	0.0%	-0.3%	-0.4%
North	Yobe	1.7%	2.7%	1.0%	0.6%	0.8%
North	Taraba	1.6%	2.4%	0.8%	0.6%	1.0%
North	Nassarawa	1.3%	1.5%	0.2%	0.4%	0.1%
North	FCT Abuja	1.0%	0.7%	-0.3%	-0.3%	-0.3%
South	Lagos	6.5%	4.2%	-2.3%	-1.1%	-2.5%
South	Oyo	4.0%	3.4%	-0.5%	-0.1%	-0.3%
South	Rivers	3.7%	2.5%	-1.2%	-1.6%	-0.6%
South	Anambra	3.0%	2.2%	-0.8%	-0.9%	-0.3%
South	Delta	2.9%	1.8%	-1.1%	-0.2%	-1.3%
South	Imo	2.8%	1.7%	-1.1%	-0.5%	-0.1%
South	Akwa Ibom	2.8%	1.7%	-1.0%	-0.3%	-1.3%
South	Ogun	2.7%	2.5%	-0.2%	0.0%	0.1%
South	Ondo	2.5%	1.5%	-1.0%	-0.3%	-0.9%
South	Osun	2.4%	1.7%	-0.8%	0.0%	-0.5%
South	Enugu	2.3%	2.1%	-0.2%	0.2%	-0.2%
South	Edo	2.3%	1.5%	-0.8%	-0.5%	-0.1%
South	Cross River	2.1%	1.6%	-0.4%	-0.4%	-0.4%
South	Abia	2.0%	1.1%	-1.0%	-0.4%	-0.8%
South	Ekiti	1.7%	0.6%	-1.1%	-1.0%	-0.9%
South	Ebonyi	1.6%	2.3%	0.7%	0.8%	0.9%
South	Bayelsa	1.2%	0.8%	-0.4%	-0.3%	-0.6%

Table W03. State weights for children age 12-23 months in DHS 2013, along with state-wise weight differences with: Census 2006, DHS 2008 and MICS 2011.

	State	Census 2006	NNHS 2014 State weight	NNHS 2014 Census 2006 Difference	NNHS 2014 No Previous NNHS	NNHS 2014 DHS 2013 Difference
North	Kano	6.7%	6.9%	0.2%		-2.1%
North	Kaduna	4.4%	5.1%	0.8%		1.4%
North	Katsina	4.1%	3.8%	-0.4%		-1.7%
North	Bauchi	3.3%	3.1%	-0.2%		-1.4%
North	Jigawa	3.1%	3.1%	0.0%		-2.2%
North	Benue	3.0%	3.1%	0.1%		-0.1%
North	Borno	3.0%	3.0%	0.1%		-0.7%
North	Niger	2.8%	2.5%	-0.3%		-1.8%
North	Sokoto	2.6%	2.6%	-0.1%		-0.9%
North	Kogi	2.4%	2.3%	-0.1%		0.9%
North	Zamfara	2.3%	2.4%	0.1%		-2.5%
North	Kebbi	2.3%	2.5%	0.2%		-1.3%
North	Plateau	2.3%	2.2%	-0.1%		0.6%
North	Adamawa	2.3%	2.3%	0.0%		0.0%
North	Kwara	1.7%	1.3%	-0.4%		0.1%
North	Gombe	1.7%	1.8%	0.1%		0.1%
North	Yobe	1.7%	2.0%	0.4%		-0.7%
North	Taraba	1.6%	1.7%	0.1%		-0.7%
North	Nassarawa	1.3%	1.3%	0.0%		-0.2%
North	FCT Abuja	1.0%	1.9%	0.9%		1.3%
South	Lagos	6.5%	6.8%	0.3%		2.6%
South	Oyo	4.0%	4.1%	0.1%		0.6%
South	Rivers	3.7%	3.7%	0.0%		1.2%
South	Anambra	3.0%	2.8%	-0.2%		0.6%
South	Delta	2.9%	2.8%	-0.2%		1.0%
South	Imo	2.8%	2.6%	-0.2%		1.0%
South	Akwa Ibom	2.8%	2.8%	0.0%		1.1%
South	Ogun	2.7%	2.7%	0.0%		0.2%
South	Ondo	2.5%	2.5%	0.1%		1.0%
South	Osun	2.4%	2.6%	0.2%		1.0%
South	Enugu	2.3%	2.2%	-0.1%		0.1%
South	Edo	2.3%	2.2%	-0.1%		0.7%
South	Cross River	2.1%	1.7%	-0.4%		0.0%
South	Abia	2.0%	1.7%	-0.3%		0.6%
South	Ekiti	1.7%	1.6%	-0.1%		1.0%
South	Ebonyi	1.6%	1.4%	-0.2%		-0.9%
South	Bayelsa	1.2%	1.0%	-0.2%		0.2%

Table W04. State weights for children age 12-23 months in NNHS 2014, along with state-wise weight differences with: Census 2006 and DHS 2013.

	State	Census 2006	NNHS 2015 State weight	NNHS 2015 Census 2006 Difference	NNHS 2015 NNHS 2014 Difference	NNHS 2015 NNHS 2014 Difference
North	Kano	6.7%	7.1%	0.4%	0.3%	0.3%
	Kaduna	4.4%	4.0%	-0.3%	-1.1%	-1.1%
	Katsina	4.1%	3.9%	-0.2%	0.2%	0.2%
	Bauchi	3.3%	3.3%	0.0%	0.2%	0.2%
	Jigawa	3.1%	3.3%	0.2%	0.2%	0.2%
	Benue	3.0%	2.9%	-0.1%	-0.2%	-0.2%
	Borno	3.0%	3.0%	0.1%	0.0%	0.0%
	Niger	2.8%	3.6%	0.8%	1.1%	1.1%
	Sokoto	2.6%	3.1%	0.4%	0.5%	0.5%
	Kogi	2.4%	2.4%	0.1%	0.2%	0.2%
	Zamfara	2.3%	2.6%	0.2%	0.2%	0.2%
	Kebbi	2.3%	2.7%	0.3%	0.2%	0.2%
	Plateau	2.3%	2.0%	-0.3%	-0.2%	-0.2%
	Adamawa	2.3%	2.4%	0.1%	0.1%	0.1%
	Kwara	1.7%	1.6%	0.0%	0.4%	0.4%
	Gombe	1.7%	1.7%	0.0%	-0.1%	-0.1%
	Yobe	1.7%	1.6%	0.0%	-0.4%	-0.4%
	Taraba	1.6%	1.4%	-0.2%	-0.3%	-0.3%
	Nassarawa	1.3%	1.2%	-0.1%	-0.1%	-0.1%
	FCT Abuja	1.0%	1.6%	0.6%	-0.3%	-0.3%
South	Lagos	6.5%	6.9%	0.4%	0.0%	0.0%
	Oyo	4.0%	3.5%	-0.5%	-0.6%	-0.6%
	Rivers	3.7%	3.7%	0.0%	0.0%	0.0%
	Anambra	3.0%	2.5%	-0.5%	-0.3%	-0.3%
	Delta	2.9%	2.7%	-0.3%	-0.1%	-0.1%
	Imo	2.8%	2.6%	-0.2%	0.0%	0.0%
	Akwa Ibom	2.8%	3.1%	0.3%	0.3%	0.3%
	Ogun	2.7%	2.5%	-0.2%	-0.2%	-0.2%
	Ondo	2.5%	2.2%	-0.2%	-0.3%	-0.3%
	Osun	2.4%	2.5%	0.1%	-0.1%	-0.1%
	Enugu	2.3%	2.3%	-0.1%	0.1%	0.1%
	Edo	2.3%	1.9%	-0.4%	-0.2%	-0.2%
	Cross River	2.1%	2.1%	0.0%	0.4%	0.4%
	Abia	2.0%	2.0%	-0.1%	0.3%	0.3%
	Ekiti	1.7%	1.6%	-0.1%	0.0%	0.0%
	Ebonyi	1.6%	1.3%	-0.3%	-0.1%	-0.1%
	Bayelsa	1.2%	1.2%	0.0%	0.2%	0.2%

Table W05. State weights for children age 12-23 months in NNHS 2015, along with state-wise weight differences with: Census 2006 and NNHS 2014.

	State	Census 2006	MICS-NICS 2016			
			State weight	Census 2006 Difference	MICS 2011 Difference	NNHS 2015 Difference
North	Kano	6.7%	8.8%	2.1%	1.8%	1.7%
	Kaduna	4.4%	6.1%	1.7%	1.7%	2.0%
	Katsina	4.1%	7.6%	3.5%	3.2%	3.7%
	Bauchi	3.3%	5.4%	2.0%	0.3%	2.1%
	Jigawa	3.1%	5.5%	2.4%	2.6%	2.2%
	Benue	3.0%	2.1%	-0.9%	-0.4%	-0.8%
	Borno	3.0%	6.5%	3.6%	3.1%	3.5%
	Niger	2.8%	4.1%	1.3%	1.3%	0.5%
	Sokoto	2.6%	3.5%	0.9%	0.3%	0.4%
	Kogi	2.4%	1.4%	-1.0%	-0.4%	-1.0%
	Zamfara	2.3%	4.9%	2.6%	2.1%	2.4%
	Kebbi	2.3%	3.2%	0.9%	0.5%	0.6%
	Plateau	2.3%	3.0%	0.8%	1.2%	1.1%
	Adamawa	2.3%	2.1%	-0.1%	-0.2%	-0.2%
	Kwara	1.7%	1.0%	-0.7%	-0.7%	-0.6%
	Gombe	1.7%	2.0%	0.3%	0.0%	0.3%
	Yobe	1.7%	4.1%	2.4%	2.2%	2.5%
	Taraba	1.6%	1.2%	-0.5%	-0.2%	-0.2%
	Nassarawa	1.3%	2.0%	0.7%	0.7%	0.8%
	FCT Abuja	1.0%	0.9%	-0.1%	-0.1%	-0.7%
South	Lagos	6.5%	4.0%	-2.5%	-2.7%	-2.9%
	Oyo	4.0%	2.5%	-1.5%	-1.3%	-1.0%
	Rivers	3.7%	1.4%	-2.3%	-1.7%	-2.3%
	Anambra	3.0%	1.2%	-1.8%	-1.3%	-1.3%
	Delta	2.9%	1.6%	-1.3%	-1.5%	-1.1%
	Imo	2.8%	1.6%	-1.2%	-0.1%	-1.0%
	Akwa Ibom	2.8%	1.9%	-0.9%	-1.1%	-1.2%
	Ogun	2.7%	1.1%	-1.6%	-1.3%	-1.4%
	Ondo	2.5%	1.5%	-1.0%	-0.8%	-0.7%
	Osun	2.4%	1.4%	-1.0%	-0.8%	-1.1%
	Enugu	2.3%	1.0%	-1.3%	-1.3%	-1.3%
	Edo	2.3%	1.0%	-1.3%	-0.6%	-0.9%
	Cross River	2.1%	1.3%	-0.8%	-0.8%	-0.8%
	Abia	2.0%	0.8%	-1.2%	-1.1%	-1.1%
	Ekiti	1.7%	0.4%	-1.3%	-1.0%	-1.2%
	Ebonyi	1.6%	0.9%	-0.6%	-0.5%	-0.4%
	Bayelsa	1.2%	0.7%	-0.6%	-0.8%	-0.6%

Table W06. State weights for children age 12-23 months in MICS-NICS 2016, along with state-wise weight differences with: Census 2006, MICS 2011 and NNHS 2015.

	State	Census 2006	NNHS 2018			
			State weight	Census 2006 Difference	NNHS 2015 Difference	MICS-NICS 2016 Difference
North	Kano	6.7%	5.9%	-0.8%	-1.2%	-2.9%
	Kaduna	4.4%	4.4%	0.1%	0.4%	-1.6%
	Katsina	4.1%	4.6%	0.4%	0.7%	-3.0%
	Bauchi	3.3%	3.3%	0.0%	0.0%	-2.0%
	Jigawa	3.1%	3.6%	0.5%	0.3%	-1.9%
	Benue	3.0%	2.7%	-0.3%	-0.2%	0.5%
	Borno	3.0%	1.4%	-1.5%	-1.6%	-5.1%
	Niger	2.8%	3.2%	0.4%	-0.4%	-0.9%
	Sokoto	2.6%	2.4%	-0.2%	-0.6%	-1.1%
	Kogi	2.4%	2.3%	-0.1%	-0.2%	0.9%
	Zamfara	2.3%	2.9%	0.6%	0.3%	-2.0%
	Kebbi	2.3%	2.6%	0.3%	-0.1%	-0.6%
	Plateau	2.3%	2.5%	0.2%	0.5%	-0.6%
	Adamawa	2.3%	2.5%	0.2%	0.1%	0.3%
	Kwara	1.7%	1.6%	-0.1%	0.0%	0.6%
	Gombe	1.7%	1.7%	0.0%	0.0%	-0.3%
	Yobe	1.7%	1.7%	0.1%	0.1%	-2.4%
	Taraba	1.6%	1.5%	-0.1%	0.1%	0.4%
	Nassarawa	1.3%	1.1%	-0.2%	-0.1%	-0.9%
	FCT Abuja	1.0%	1.6%	0.6%	0.0%	0.8%
South	Lagos	6.5%	6.6%	0.1%	-0.3%	2.6%
	Oyo	4.0%	4.1%	0.1%	0.6%	1.6%
	Rivers	3.7%	3.8%	0.1%	0.1%	2.4%
	Anambra	3.0%	3.3%	0.3%	0.8%	2.1%
	Delta	2.9%	2.7%	-0.2%	0.1%	1.1%
	Imo	2.8%	2.4%	-0.4%	-0.2%	0.8%
	Akwa Ibom	2.8%	3.1%	0.3%	0.0%	1.2%
	Ogun	2.7%	2.7%	0.1%	0.2%	1.6%
	Ondo	2.5%	2.4%	-0.1%	0.2%	0.9%
	Osun	2.4%	2.5%	0.1%	0.0%	1.1%
	Enugu	2.3%	2.4%	0.1%	0.1%	1.4%
	Edo	2.3%	2.1%	-0.2%	0.2%	1.1%
	Cross River	2.1%	2.0%	0.0%	-0.1%	0.7%
	Abia	2.0%	2.0%	0.0%	0.1%	1.2%
	Ekiti	1.7%	1.8%	0.1%	0.2%	1.4%
	Ebonyi	1.6%	1.3%	-0.3%	0.0%	0.4%
	Bayelsa	1.2%	1.0%	-0.2%	-0.2%	0.4%

Table W07. State weights for children age 12-23 months in NNHS 2018, along with state-wise weight differences with: Census 2006, NNHS 2015 and MICS-NICS 2016.

	State	Census 2006	DHS 2018			
			State weight	Census 2006 Difference	DHS 2013 Difference	NNHS 2018 Difference
North	Kano	6.7%	7.0%	0.3%	-1.9%	1.1%
North	Kaduna	4.4%	5.9%	1.5%	2.1%	1.5%
North	Katsina	4.1%	6.4%	2.2%	1.0%	1.8%
North	Bauchi	3.3%	4.5%	1.2%	0.0%	1.2%
North	Jigawa	3.1%	4.0%	0.9%	-1.3%	0.4%
North	Benue	3.0%	3.2%	0.1%	-0.1%	0.5%
North	Borno	3.0%	3.6%	0.6%	-0.2%	2.2%
North	Niger	2.8%	4.2%	1.3%	-0.2%	1.0%
North	Sokoto	2.6%	2.9%	0.3%	-0.5%	0.5%
North	Kogi	2.4%	1.3%	-1.1%	-0.1%	-1.0%
North	Zamfara	2.3%	3.6%	1.2%	-1.4%	0.7%
North	Kebbi	2.3%	3.4%	1.1%	-0.4%	0.8%
North	Plateau	2.3%	1.7%	-0.6%	0.1%	-0.8%
North	Adamawa	2.3%	2.5%	0.2%	0.2%	0.0%
North	Kwara	1.7%	1.7%	0.0%	0.5%	0.1%
North	Gombe	1.7%	2.1%	0.4%	0.4%	0.4%
North	Yobe	1.7%	3.6%	1.9%	0.9%	1.9%
North	Taraba	1.6%	2.2%	0.5%	-0.2%	0.7%
North	Nassarawa	1.3%	1.4%	0.1%	-0.1%	0.3%
North	FCT Abuja	1.0%	0.7%	-0.3%	0.0%	-1.0%
South	Lagos	6.5%	5.4%	-1.1%	1.1%	-1.3%
South	Oyo	4.0%	2.9%	-1.1%	-0.5%	-1.2%
South	Rivers	3.7%	2.8%	-0.9%	0.3%	-1.0%
South	Anambra	3.0%	3.6%	0.6%	1.4%	0.3%
South	Delta	2.9%	2.1%	-0.9%	0.2%	-0.7%
South	Imo	2.8%	1.9%	-0.9%	0.2%	-0.5%
South	Akwa Ibom	2.8%	1.9%	-0.9%	0.2%	-1.2%
South	Ogun	2.7%	2.0%	-0.7%	-0.6%	-0.8%
South	Ondo	2.5%	1.2%	-1.2%	-0.3%	-1.2%
South	Osun	2.4%	1.7%	-0.8%	0.0%	-0.8%
South	Enugu	2.3%	1.4%	-0.9%	-0.7%	-1.0%
South	Edo	2.3%	1.2%	-1.1%	-0.3%	-0.9%
South	Cross River	2.1%	0.9%	-1.2%	-0.8%	-1.2%
South	Abia	2.0%	1.1%	-0.9%	0.1%	-0.9%
South	Ekiti	1.7%	1.1%	-0.6%	0.6%	-0.7%
South	Ebonyi	1.6%	2.4%	0.9%	0.1%	1.1%
South	Bayelsa	1.2%	0.8%	-0.4%	0.0%	-0.2%

Table W08. State weights for children age 12-23 months in DHS 2018, along with state-wise weight differences with: Census 2006, DHS 2013 and NNHS 2018.

5 Geographic Variability in National Centroids of State Weights

Figure 2 in the main paper shows that northern states received substantially more weight in MICS/NICS 2016-17 than NNHS 2015. This section of the supplement portrays the aggregate north-south and east-west components of state weights from the ten surveys in question by calculating a weighted average of 37 state-level geographic centroids.

5.1 Methods

The inputs to the analysis are survey state weights (based on children aged 12–23 months) and geo-coordinate of the spatial centroid of each of Nigeria's 37 states.

For survey k, the weight for state i is calculated as described in the main paper:

$$SW_{ik} = \frac{\sum_{j=1}^{n_{ik}} w_{ijk}}{\sum_{i=1}^{37} \sum_{j=1}^{n_{ik}} w_{ijk}}$$

where i indexes over 37 states and j indexes over the n_{ik} respondents in state i in survey k, and w_{ijk} is the weight assigned to respondent j in state i in survey k.

State centroid coordinates Lat_i and Lon_i were calculated using a GIS shapefile of state boundaries and a user-written Stata [1] command named shp2dta [2]. For each of the ten surveys, the age 12–23 months population-weighted national centroid LAT_k and LON_k was calculated using the state weights and centroids:

$$LAT_k = \frac{\sum_{i=1}^{37} SW_{ik} Lat_i}{\sum_{i=1}^{37} SW_{ik}}$$

$$LON_k = \frac{\sum_{i=1}^{37} SW_{ik} Lon_i}{\sum_{i=1}^{37} SW_{ik}}$$

5.2 Results

Figure S1 shows the national centroid coordinates alone (panel a) and with labels (panel b). All three NNHS centroids cluster closely together because of post-stratification. MICS 2007 and 2011 fall just northeast of the NNHS centroids. DHS centroids are clustered slightly farther northeast. The MICS/NICS 2016-17 centroid is substantially farther north and somewhat farther east than any of the other nine surveys.

a. Unlabeled Centroids

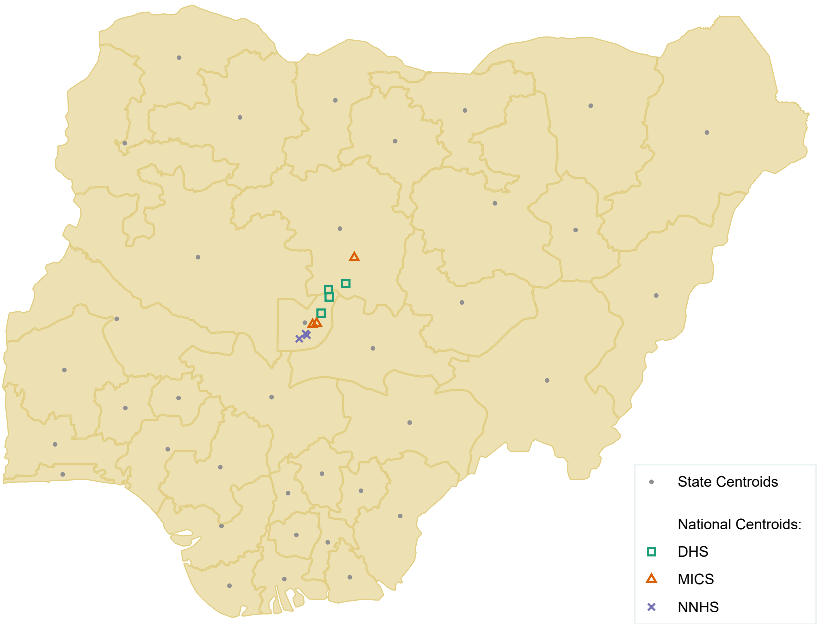

b. Labeled by Year

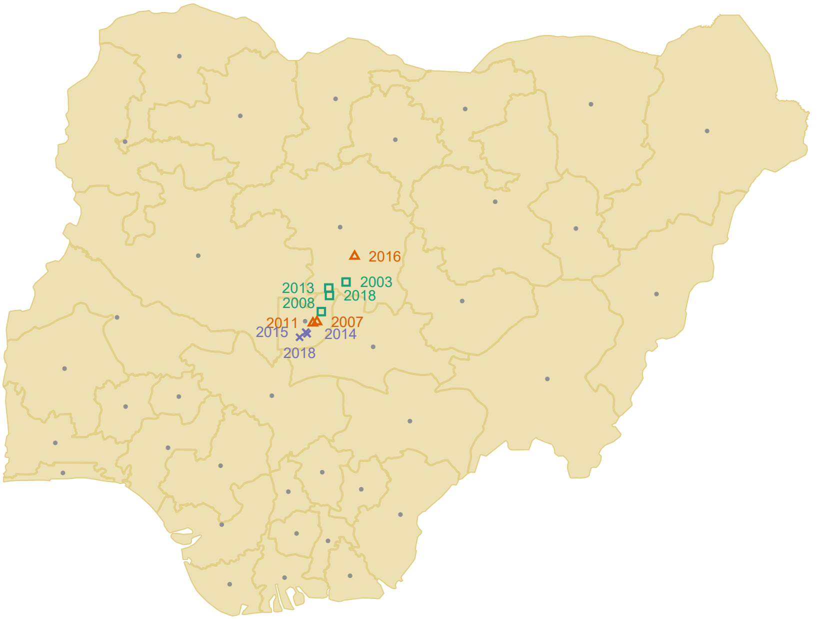

Figure S1: National Centroids of State Weights from all Ten Surveys

5.3 Discussion

The 2016 northward shift in state weights amplified the well-documented north-south disparity in vaccination coverage outcomes, giving the appearance of a double-digit drop in DPT3 and MCV1 from MICS 2011 to MICS/NICS 2016-17, and a double-digit drop in DPT3 when examining the apparent difference from NNHS 2015 to MICS/NICS 2016-17. If the MICS/NICS 2016-17 had used state weights more like those from the 2006 census, or more like those from any of the other nine surveys considered here, the apparent drop in coverage would have been smaller.

References

[1] StataCorp. Stata Statistical Software: Release 16, 2019. College Station, TX.

[2] Kevin Crow and William Gould. How do I graph data onto a map with spmap?, 2015. Stata FAQ. `https://www.stata.com/support/faqs/graphics/spmap-and-maps`. [Online, accessed on 2020-04-27].
